# Traditional Chinese Medicine Integrated Multifunctional Responsive Core–Shell Microneedles for Dermatosis Treatment

**DOI:** 10.34133/research.0420

**Published:** 2024-07-04

**Authors:** Xi Luan, Xiaoxuan Zhang, Qichen Luan, Jingjing Gan, Yu Wang, Yuanjin Zhao

**Affiliations:** ^1^ Department of Rheumatology and Immunology, Nanjing Drum Tower Hospital, School of Pharmacy, Clinical College of Traditional Chinese and Western Medicine, Nanjing University of Chinese Medicine, Nanjing 210023, China.; ^2^ State Key Laboratory of Bioelectronics, School of Biological Science and Medical Engineering, Southeast University, Nanjing 210096, China.; ^3^ Shenzhen Research Institute, Southeast University, Shenzhen 518071, China.

## Abstract

Microneedles have demonstrated value in targeted treatment of dermatosis. Current investigation aims to enhance the functions and optimize substance delivery to improve therapeutic effects. Here, we present innovative shell–core microneedles with light-pH dual responsiveness for spatiotemporal sequential release of multiple Chinese herb drugs to treat scleroderma. By using a stepwise template-assisted method, we effectively prepare a hydrogel-based core layer containing polydopamine-MXene (P-MXene) loaded with triptolide (TP), and a shell layer composed of polyvinyl alcohol (PVA) encapsulating paeoniflorin (Pae). P-MXene can adsorb the sparingly soluble TP to ensure its encapsulation efficiency and contribute to the synergistic photothermal effect benefitting from its excellent photothermal conversion ability. Besides, PVA can rapidly dissolve upon microneedle piercing into the skin and quickly release the anti-inflammatory and detoxifying Pae, establishing a favorable low-acid subcutaneous environment. In response to pH changes and near-infrared effects, TP is sustainably released from P-MXene and delivered through the swollen pores of the hydrogel. On the basis of these characteristics, we demonstrate that these microneedles could effectively reduce profibrotic key cytokines interleukin-1β and transforming growth factor-β, thereby reducing collagen deposition and decreasing epidermal thickness, ameliorating skin fibrosis and capillary lesion in scleroderma mouse models. These findings highlight the important clinical potential of these microneedles in the treatment of skin diseases.

## Introduction

Dermatological disorders have long plagued mankind, causing significant distress and negatively affecting the overall quality of life [1]. Among these disorders, skin lesions resulting from intractable diseases such as scleroderma present a particularly daunting treatment challenge [2–4]. In addition to commonly used treatment strategies of systemic drug administration, topical drug application has gained prominence as a more desirable and promising therapeutic option due to the directness, precision, and reduced side effects [5,6]. To realize this, microneedles (MNs) have been developed, which are micrometer-sized needles capable of penetrating the stratum corneum and allowing the targeted treatment of skin diseases [7,8]. These MNs can carry a diverse range of active ingredients, making them a versatile platform for treating various dermatological conditions [9,10]. Notably, functional materials such as graphene oxide and black phosphorus can be integrated into MNs to facilitate controlled release, yielding specific therapeutic outcomes [11–15]. Despite these advancements, current research on MNs for skin diseases still faces challenges [16]. One major obstacle lies in the uniform structure of most current MNs, which significantly hinders temporally and spatially controlled release of medications [17,18]. In addition, the effectiveness of conventional small-molecule medications remains unsatisfactory [19]. Therefore, there is a pressing need to develop innovative MN delivery devices for skin disease treatment [19–21].

**Fig. 1.**
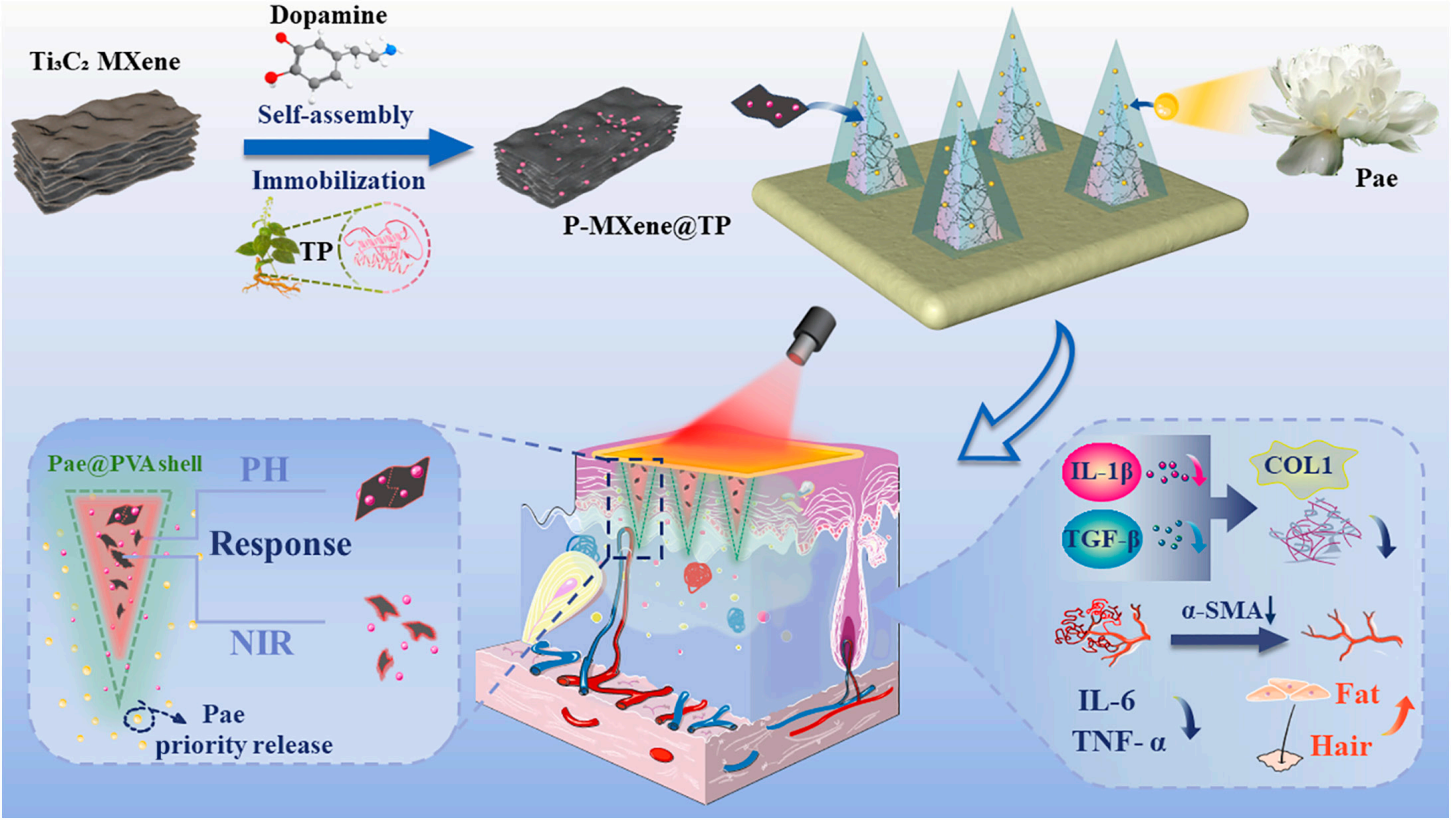
Schematic illustrations of multiresponse core–shell MNs loaded with TP and Pae for the treatment of early skin lesions of scleroderma.

Here, we proposed a light-pH dual-responsive MN system with a shell–core structure encapsulating multicomponent herbal drugs to achieve spatiotemporal sequential release for scleroderma treatment, as illustrated in Fig. 1. Triptolide (TP) and paeoniflorin (Pae) are known for their anti-inflammatory and immune regulatory effects, making them promising candidates for treating scleroderma [22–25]. To facilitate effective delivery, the drugs were loaded on different layers of the multifunctional shell–core MNs by a stepwise template-assisted method. TP was encapsulated in the core layer together with polydopamine (PDA)-modified Ti_3_C_2_ MXene (denoted as P-MXene), which could adsorb the sparingly soluble TP [26]. PDA played a crucial role as a protective barrier, effectively shielding MXene from oxidation and degradation [27], while it actively contributed to the synergistic photothermal effect [28,29], leveraging its exceptional photothermal conversion capability [30]. The shell layer, carrying Pae and supported by polyvinyl alcohol (PVA), could rapidly dissolve upon MN piercing into the skin and quickly release Pae. Pae, which targets the liver meridian, has properties that can soften and protect the liver. It plays a significant role in alleviating the liver and kidney damage that may be caused by TP. The precursor release of Pae provided an anti-inflammatory, soothing, and detoxifying low-acid environment, leading to sustained release of TP from P-MXene in the low-acid environment and upon photothermal conversion. The robust anti-inflammatory and immunomodulatory effects of these MNs were further demonstrated in the treatment of scleroderma in mouse models [31], indicating their practical and potential values.

## Results and Discussion

### Morphology characterization of core–shell MNs

**Fig. 2.**
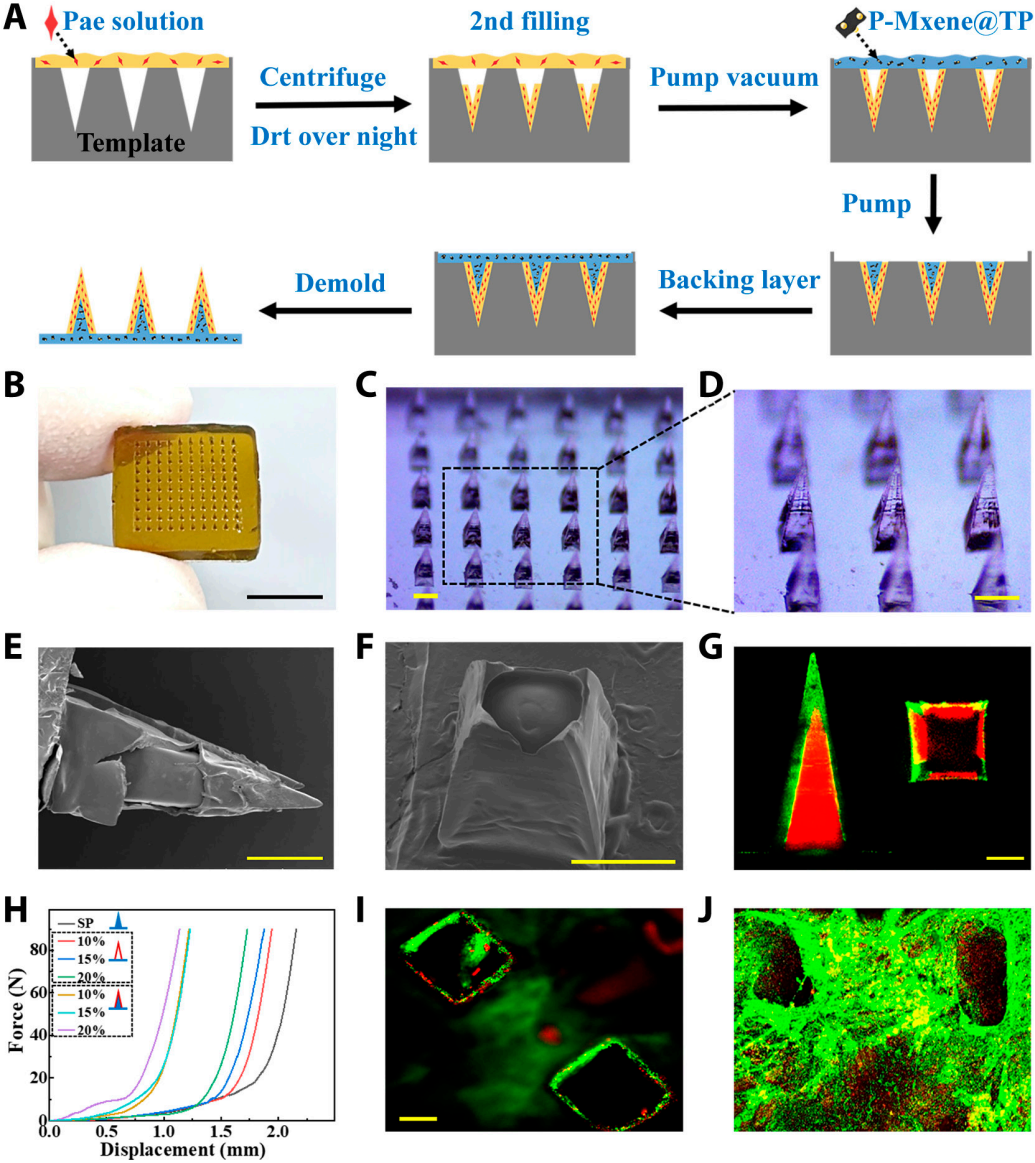
Characterization and mechanical strength of the MNs. (A) Schematic illustration of the fabrication of the multiresponse MNs. (B) Optical image of the core–shell MN patch. (C and D) Microscopy image of the MN array. (E and F) SEM images of the shell–core structure MNs. (G) Confocal images of MNs loaded with fluorescent nanoparticles to characterize the shell–core structure of tips. (H) The forces of the MNs with different structures and compositions. (I) Confocal image of the appearance of the porcine dermatome after MN puncture. (J) Subcutaneous infiltration of the skin’s inner layer after 6 h of MN penetration of porcine skin with dual-colored nanoparticles. Scale bars, 500 μm.

Typically, we used a template-assisted curing method to craft core–shell MNs through sequential material filling (Fig. 2A). The outer layer of the MNs was composed of mechanically robust and water-soluble PVA, infused with the active pharmaceutical ingredient Pae, and the acidic environment created by dissolving PVA is just right to help keep Pae’s properties stable. This composite material was introduced into the mold, followed by drying at 37 °C overnight. This process was iterated to ensure uniform distribution of the desiccated PVA shell within the mold, culminating in the formation of the tip. Subsequently, a composite material composed of sodium alginate (SA) and polyethylene glycol diacrylate (PEGDA) encapsulating P-MXene@TP (P-M@TP) was rapidly introduced into the PVA-pretreated mold using a vacuum and then cured under ultraviolet light. The ultimate step involved demolding the structure to yield a complete MN patch (Fig. 2B). This architecture facilitated distinct and staged MN release with varying efficiencies. The resulting MNs exhibited a prismatic cone array configuration, measuring 1,000 μm in height (Fig. 2C and D). Scanning electron microscopy (SEM) imaging meticulously elucidated the intricate shell–core morphology of the MNs from diverse viewpoints (Fig. 2E and F). To explore the intricate structure and the distribution of 2 distinct drugs within the MNs, we used fluorescent nanoparticles in 2 distinct colors. These nanoparticles simulated the inner and outer layers of the drugs, respectively. Visualization using laser confocal microscopy provided insights into the spatial distribution of the drugs within the MNs (Fig. 2G). The core–shell architecture triggered the initial release of the pioneer drug through a rapid dissolution of the outer layer, simultaneously fostering a mildly acidic microenvironment within the inner layer that aided in carrier activation.

The mechanical characteristics of the bilayer MN array vary in response to different concentrations of support material. The presence of PVA in the outer shell, combined with the reinforcing effects of the inner hydrogel layer, enhances the MNs’ ability to efficiently penetrate the skin. To assess mechanical strength, we conducted experiments using a universal material testing machine. As the mechanical sensor element gradually applied downward pressure, the change in force on the MN over time was recorded. The recording commenced upon contact with the array tip, and measurements ceased upon reaching a load capacity of 90 N. Our findings reveal a direct correlation between the mechanical strength of the MNs and the concentration of PVA. Notably, the incorporation of a double-layer structural design led to a substantial increase in mechanical resilience (Fig. 2H). Stress–displacement curves illustrate that single-needle MNs can endure forces exceeding 0.9 N, sufficient for successful skin penetration. The morphology of the tips before and after compression was shown in Fig. S1. The delicate balance between mechanical strength and the outer shell’s solubility guided our choice of materials. Specifically, the outer shell was composed of 20% PVA, while the inner shell consisted of 4% SA-PEGDA. Fluorescent nanoparticles of various colors were incorporated into the shell and core layers to simulate the 2 drugs loaded in the MNs. These fabricated MNs underwent puncture experiments on the porcine skin to simulate the drug release process at 37 °C. Laser confocal microscopy revealed clear penetration channels on the porcine skin after the MN patches were removed following 6 h of application (Fig. 2I). Layer-by-layer scanning of the pig skin demonstrated that the green nanoparticles, representing Pae in the outer layer, fully penetrated all skin layers within 6 h. Conversely, the red nanoparticles in the inner layer, simulating TP, were only partially released into the skin, indicating a controlled release state in the MN design (Fig. 2J).

### Synthesis and characterization of nanoparticles

**Fig. 3.**
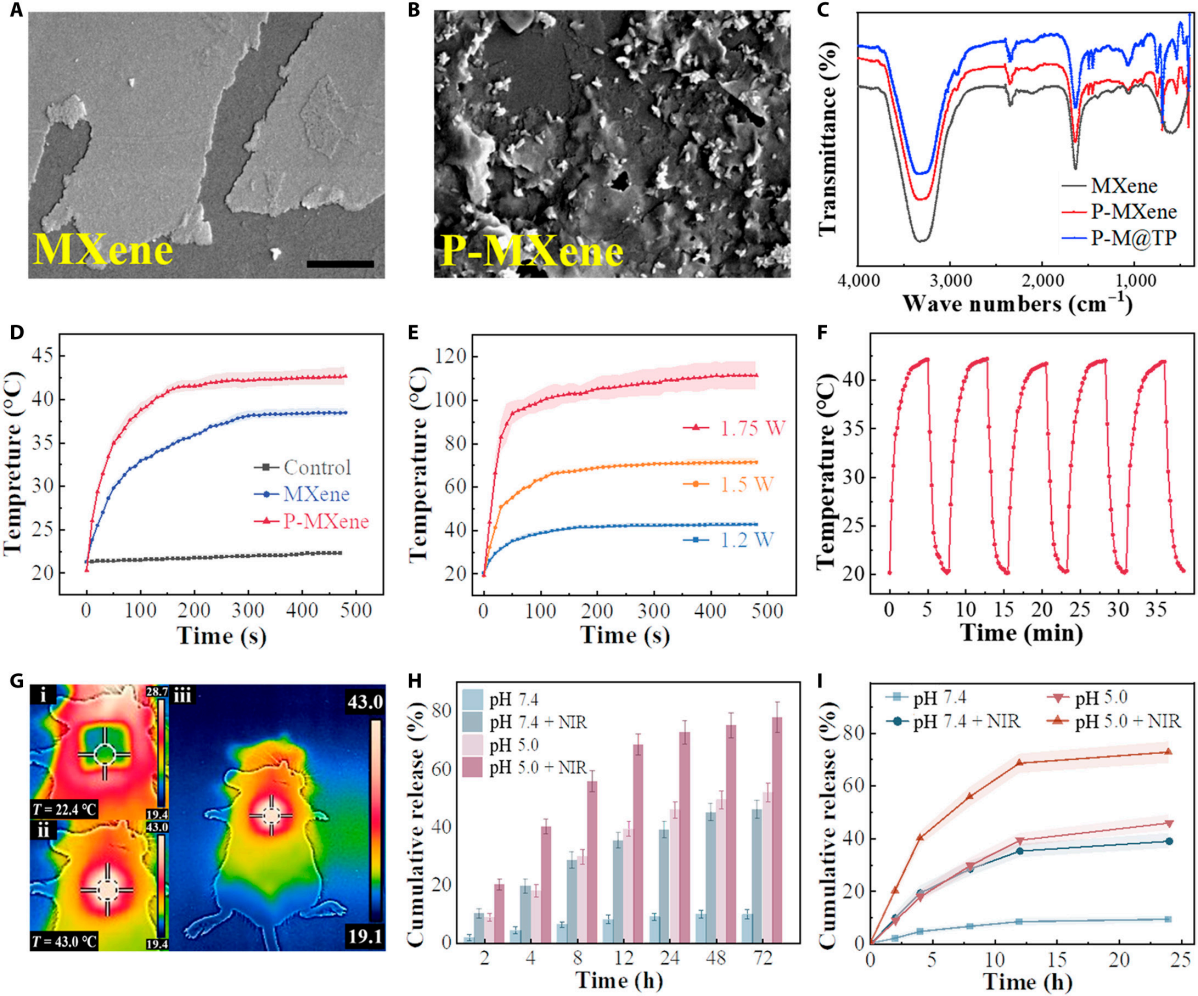
Photothermal properties of P-MXene and sensitivity release to NIR and pH. (A and B) SEM images of the MXene and P-MXene. (C) Fourier transform infrared spectra of MXene, P-MXene, and P-M@TP. (D) Photothermal heating curves of MNs encapsulating MXene and P-MXene. (E) Temperature rising profiles of the MNs under NIR light of 1.2, 1.5, and 1.75 W. (F) Recycling heating profiles of P-M@TP MNs during 5 on/off cycles. The power of NIR light was 1.2 W. (G) Thermal images of MNs applied to the mouse dorsal skin before and after 2-min NIR irradiation. The NIR power was 1.2 W. (H and I) In vitro drug release profiles from P-M@TP MNs in PBS at pH 7.4 and 5.0 and about the availability of 808-nm laser stimulation.

To validate the synthesis of self-assembled P-MXene nanoparticles, we conducted a comprehensive characterization. Figure 3A illustrated the morphology of MXene before processing, displaying smooth lamellae when viewed under the electron microscope. In contrast, Fig. 3B and Fig. S2 depicted the postsynthesis state, demonstrating the encapsulation of PDA around the MXene surface. This structural modification exhibited the ability to mitigate the oxidation of MXene to some extent. In addition, our investigation revealed the long-term stability and homogeneity of the synthesized nanoparticle system (Fig. S3). Furthermore, we explored the utility of the PDA outer layer in these nanoparticles as a refractory carrier for therapeutic payloads. Specifically, we used the water-insoluble chemical Nile Red to evaluate the system’s ability to carry drugs. Remarkably, our results indicated a drug loading rate approaching 80% within a 4-h period (Fig. S4). To confirm the successful synthesis of these nanoparticles, we used infrared spectroscopy to analyze MXene, P-MXene, and P-M@TP (Fig. 3C). The infrared spectra unveiled distinctive peaks at 3,330.91, 1,635.49, 1,395.73, and 585.12 cm^−1^, corresponding to the stretching vibrations of the –OH, C, O, O–H, and Ti–O bonds in MXene, respectively. These observations aligned with previous report [27]. Notably, we observed a blueshift in P-MXene and P-M@TP, with the peak shifting from 585.12 to 539.45 cm^−1^ in comparison to pristine MXene. This shift is caused by the interaction of PDA and TP with MXene. Furthermore, Fourier transform infrared spectra indicated that PDA and TP were successfully doped within the MXene framework.

### Performance of photothermal conversion

The synthesized P-MXene exhibited photothermal capabilities when exposed to a specific near-infrared (NIR) wavelength band. To investigate this property, we conducted tests using MXene and PDA-modified nanoparticles at a power level of 1.2 W (Fig. 3D). These experiments revealed that the modification of MXene by PDA enhanced its ability to convert NIR illumination at 808 nm into a higher temperature increase. P-MXene displayed distinct photothermal properties at various concentrations controlled by high and low power (Fig. 3E). Notably, when subjected to 1.2-W NIR power, the MNs rapidly reached temperature around 42 °C within 3 min. Furthermore, the maximum temperature of the MNs increased with the elevation of NIR power. At 1.5 W, the maximum temperature reached 71 °C, and at 1.75 W, the MN temperature quickly reached 110 °C in 2 min. However, it is crucial to acknowledge that both higher NIR power and elevated temperatures can potentially harm the skin. Therefore, on the basis of these results, we determined that 1.2 W is the optimal condition for our experiments. We implemented consecutive NIR switching cycles in succession on the MN to determine whether this capability of conversion could be consistently activated by NIR light. Throughout these cycles, we observed that the MN’s ability to switch between heating and cooling remained consistently effective, as illustrated in Fig. 3F. According to our findings, the photothermal transformation procedure for MNs can be reliably repeated with NIR control. Our research demonstrated that MNs not only generated temperature when activated with the NIR in vitro but can also be effectively used to the skin of mice. We found a rapid increase in the localized skin temperature of mice using a thermal imaging camera, rising from 22 to approximately 43 °C in 2 min, thereby validating the efficacy of in vivo photothermal conversion (Fig. 3G).

### Drug-responsive release behavior of MNs

PDA disintegrates in a mildly acidic environment, releasing the drug it carries. To investigate the acid-triggered drug release mechanism from the inner core layer to the dissolution of the outer PVA layer, we conducted experiments on drug release from P-M@TP under different conditions, both with and without NIR laser irradiation. For the sake of simulated release experiments, we substituted TP with curcumin (Cur) due to its similar physicochemical properties. Two sets of MNs composed solely of the inner core layer structure were submerged separately. One set was immersed in phosphate-buffered saline (PBS) buffer with a pH of 7.4, while the other was placed in PBS buffer containing an equivalent amount of PVA dissolved in the outer shell layer of the MNs. The pH of the solution with the dissolved outer shell layer material was determined to be approximately 5.0. In the absence of NIR irradiation, after 72 h, only 10% of Cur was released at pH 7.4. In contrast, at pH 5.0, 52.1% of Cur was released (Fig. 3H), highlighting P-M@TP’s sensitivity to the acidic microenvironment created by PVA dissolution. The quantity of released drug increased, and the release rate was notably accelerated when exposed to an 808-nm NIR laser (at a rate of 1.25 W/cm per pulse for 5 min). Cumulatively, the release of Cur increased from 10.1% to 45.9% at pH 7.4 and from 52.1% to 77.8% at pH 5.0 (Fig. 3I). The application of NIR light significantly expedited the release rate, offering a rapid and controlled NIR laser-triggered drug release mechanism. This feature facilitated the accumulation of high drug concentrations at the lesion site while mitigating the negative effects of TP through the preferential release of Pae.

### Biocompatibility of MNs and synergies between drugs

**Fig. 4.**
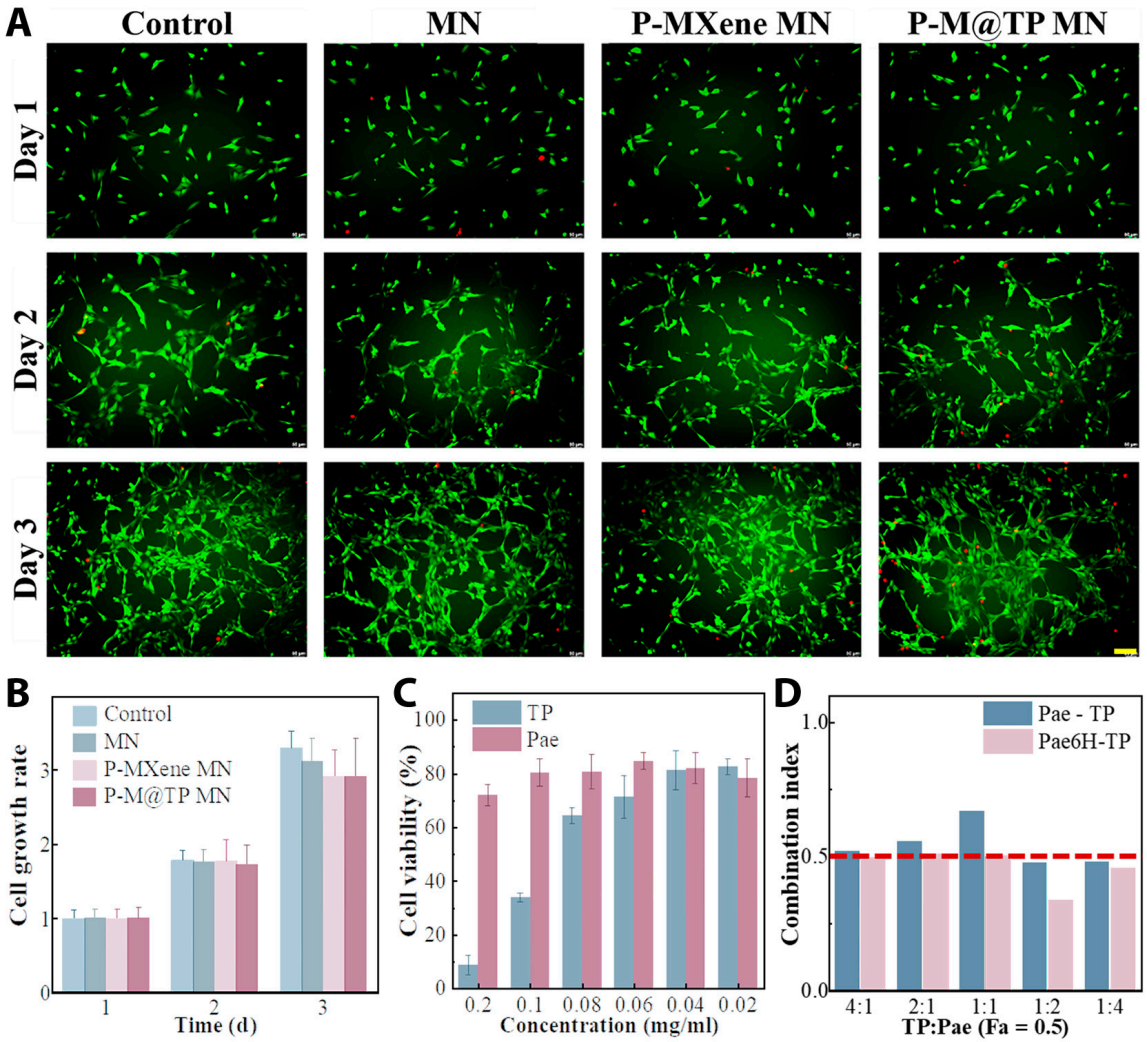
Biocompatibility of the MNs, efficacy of drugs, and synergy between 2 drugs. (A) NIH-3T3 cells cocultured with MN, P-MXene MN, and P-M@TP+Pae (P-M@TP) at days 1, 2, and 3. Scale bar, 100 μm. (B) Cell growth rate of NIH-3T3 cells cocultured with materials for 3 d. (C) Cell viability after cocultured with different concentrations of TP and Pae, respectively. (D) The CI values at Fa = 0.5 of different combinations and administration modes of TP with Pae (CI > 1 indicates antagonism, CI < 1 is the synergistic effect, and CI < 0.5 is the strong synergistic effect).

To ensure the safety and biocompatibility of the MNs, we conducted various experiments using standard fibroblast cell lines (3T3). These experiments included the evaluation of a blank well plate group without MN (control), a group with the blank MN patch with only the material (MN), a group with the addition of P-MXene (P-MXene MN), and a group with P-M@TP+Pae (P-M@TP MN). The results confirmed that all components were safe and biocompatible (Fig. 4A and B). To assess drug toxicity, we utilized a human dermal fibroblast cell line to study the interaction and toxicity of the drugs, TP and Pae. We applied the Cell Counting Kit-8 (CCK-8) method to evaluate cell viability under various TP and Pae concentrations (Fig. 4C). To further understand the mode of action of these 2 medicines, we used the CCK-8 method to quantify the strength and quality of their interaction at different concentration ratios. In addition, we simulated the interaction effect of the 2 drugs concerning layered release versus simultaneous release. We incubated both drugs simultaneously for 12 h in one group, while in the other group, we simulated a sequential release pattern by initially incubating Pae for 6 h, followed by the addition of TP and another 6 h of incubation. A combined index (CI) was used for quantitative analysis, where CI values below 0.5 indicate strong synergy and a smaller CI value indicates a stronger synergistic effect. In the results shown by Fa-CI (Fa: inhibition rate) in Fig. 4D and Fig. S5, the sequential dosing group all exhibited strong synergistic effects between the 2 drugs across a ratio range of 4:1 to 1:4, therein the strongest synergistic effect occurred at a TP:Pae ratio of 1:2. This combination also resulted in preferential Pae release for 6 h, enhancing the overall synergistic effect.

### Efficacy validation on scleroderma mouse models

**Fig. 5.**
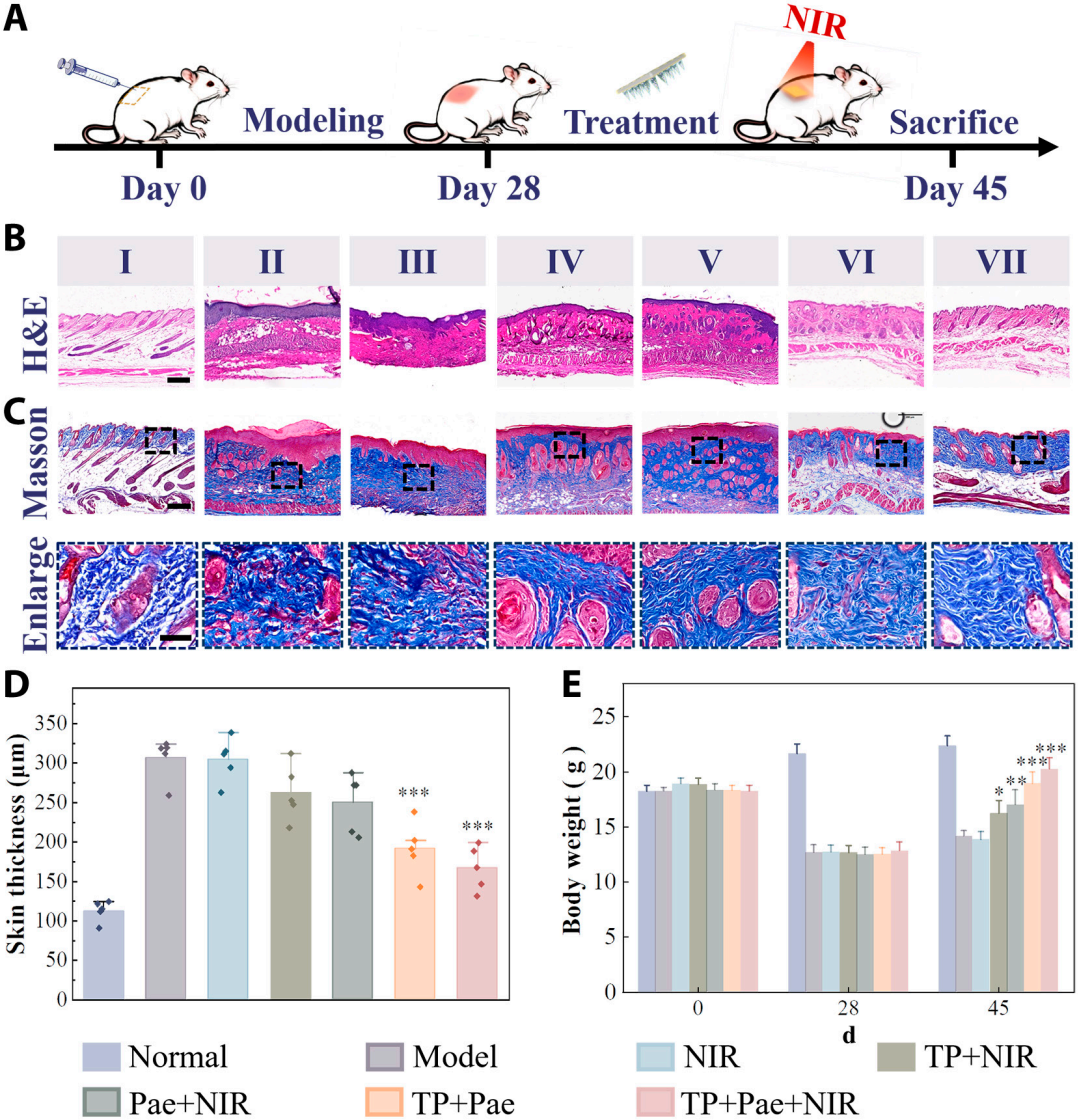
Model establishment and therapeutic effect of the MN patches on scleroderma model mice. (A) Process of the establishment and treatment of the scleroderma mouse model. (B) Corresponding H&E staining of the skin lesions of different groups on day 45. (C) Collagen deposition illustrated by Masson’s trichrome staining of different groups on day 45. (D) The skin thickness of the mice in corresponding groups. All statistical differences were compared with the model group. (E) Body weight of the corresponding groups at different stages during the induction and therapy of bleomycin-induced scleroderma model mice. Statistical differences were compared with the corresponding groups on day 28. (I: normal group; II: model group; III: NIR group; IV: TP+NIR group; V: Pae+NIR group; VI: TP+Pae group; VII: TP+Pae+NIR group). Scale bars, 200 μm. *P < 0.05, **P < 0.01, and ***P < 0.001.

To substantiate the practical efficacy of MNs, dermal sclerosis was induced through repeated subcutaneous injections of bleomycin over a 28-d period. Detailed methods are available in previous report [31]. After 28 d, histopathological examination revealed definitive dermal sclerosis, characterized by collagen bundle thickening and homogeneous deposition of thickened dermal material with cellular infiltration—resembling histological features of human scleroderma. Successfully modeled mice were randomly assigned to 6 groups. Before modeling, a negative reference group was left raw, and a positive response group was established (Fig. 5A). The remaining 5 groups were treated with various MNs: blank MNs with drug removal but NIR exposure (NIR group), MNs with TP added and simultaneous NIR irradiation (TP+NIR group), MNs with Pae added and simultaneous NIR irradiation (Pae+NIR group), MNs with addition of TP and Pae without NIR stimulation (TP+Pae group), and MNs with addition of TP and Pae with NIR intervention (TP+Pae+NIR group).

Hematoxylin and eosin (H&E) staining illustrated changes in overall skin layer thickness (Fig. 5B and D), with the positive control group exhibiting nearly twice the normal skin thickness. Notably, there was a significant reduction in skin thickness following treatment. Masson staining showcased collagen arrangement regularization and reduced deposition after drug-containing MN treatment (Fig. 5C). Proline content analysis indicated that the group of 2 drugs combined under NIR stimulation reduced hydroxyproline content, correlating with fibrotic lesion severity, thereby improving skin fibrosis (Fig. S6). To assess MN toxicity, weight changes of mice were monitored. Postmodeling, where there was a significant reduction in weight, largely restored after the 2-drug MN combination treatment (Fig. 5E). The 2-drug combination group of mice exhibited the best recovery of body weight, while the body weight of mice treated with TP alone remained relatively low. Organ staining and weighing confirmed the minimal toxicity of the composite herbal MNs to mouse organs (Figs. S7 and S8). In addition, the organ indices suggested that the 2-drug combination group was less toxic than TP alone. Minimal organ toxicity was observed in the 2-drug combination MN group during drug administration.

**Fig. 6.**
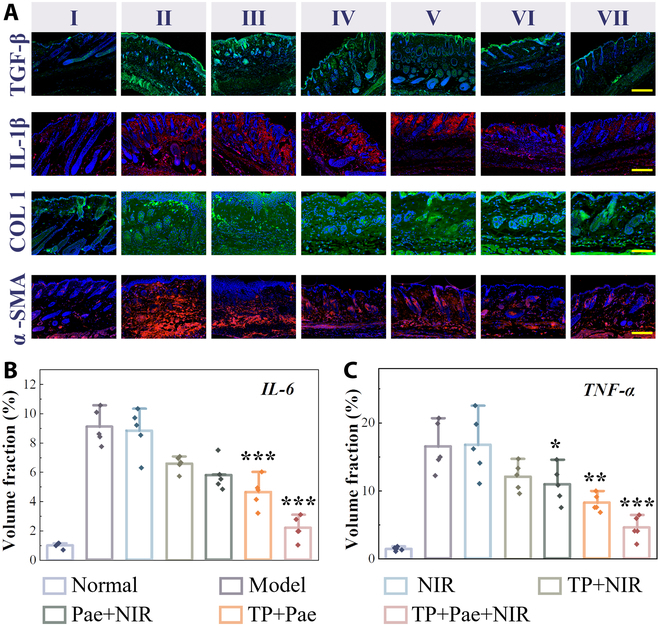
Investigation of the biological mechanism of the scleroderma mice. (A) The expression of TGF-β, IL-1β, COL1A, and α-SMA was detected by immunofluorescent staining in different groups. (B and C) Fluorescence volume fraction statistics of IL-6 and TNF-α (I: normal group; II: model group; III: NIR group; IV: TP+NIR group; V: Pae+NIR group; VI: TP+Pae group; VII: TP+Pae+NIR group). Scale bars, 200 μm. All statistical differences were compared with the model group.

Further elucidating the lesion repair process and biological mechanism, immunofluorescence staining for key profibrotic cytokines [transforming growth factor-β1 (TGF-β1) and interleukin-1β (IL-1β)] revealed significant increases in scleroderma model mice, reduced by the TP+Pae+NIR group after treatment (Fig. 6A). Collagen type I alpha 1 (COL1A1) expression and type I collagen deposition were notably decreased in this group (Fig. 6A). α-Smooth muscle actin (α-SMA)-positive myofibroblasts, prevalent in bleomycin-induced scleroderma, decreased with MN treatment. Inflammatory factors IL-6 and tumor necrosis factor-α (TNF-α) were effectively suppressed by the TP+Pae+NIR group (Fig. 6A to C and Fig. S9). Increased perilipin 1 (PLIN1) expression suggested fat layer generation in the combined treatment group (Fig. S10). Given its superior performance in each parameter, the TP+Pae+NIR group emerged as the most effective treatment. Notably, other drug-independent parameters, such as NIR or MN patches alone, had minimal therapeutic effects on scleroderma. These findings underscored the effectiveness of the multiple-response combined drug delivery MN system in treating early scleroderma skin injury.

## Conclusion

In conclusion, we propose a novel approach to topical drug delivery through photoresponsive and pH dual-responsive patches loaded with TP and Pae. These patches facilitate controlled spatiotemporal sequential release of drugs, offering a potential solution for recalcitrant skin injuries. The global urgency in addressing recalcitrant skin diseases stems from their intractability and high recurrence, imposing significant burdens on afflicted patients. Current clinical drug applications are constrained by elevated adverse reactions, limited potency, intense side effects, and challenges in precise drug administration. Consequently, there is a critical need to explore alternative therapies that provide safe and precise localized treatment to alleviate patient suffering. Therefore, we designed a shell and nucleus structured multiresponsive herbal compound MN for treating scleroderma skin lesions. The outer layer of MN, composed of PVA with excellent solubility and mechanical strength, supported Pae. The inner layer, a hydrogel, encapsulated TP carried by P-MXene, known for its outstanding immunomodulatory capabilities and effectiveness in minimizing TP’s side effects. The initial release of Pae from the outer layer established a local anti-inflammatory and detoxification microenvironment on the skin. Subsequently, TP, which regulated immune and anti-inflammatory responses, was slowly released through controlled mechanisms. The synergistic interaction of TP and Pae proved particularly beneficial in modulating the immune system, improving skin fibrosis, and reducing inflammation. P-MXene, with its robust photothermal effect and capacity to carry TP, induced rapid temperature elevation under NIR irradiation. The weak acidic environment resulting from PVA dissolution in the outer layer of MNs triggered the release of TP from PDA. Importantly, our MNs exhibited promising utility in treating early whole skin wound models in scleroderma mice, indicating their potential for broad application in intractable dermatology and related biomedical domains.

## Materials and Methods

### Characterization

The morphology of MNs was examined using a Nikon orthophoto microscope for bright-field observation. SEM (HITACHI SU8010) was used to obtain SEM images of nanoparticles. Fluorescence microscopy (Olympus, IX73-A12FL/PH) was utilized for observing fluorescence photographs. Zeiss laser scanning confocal microscope (LSM 980 with Airyscan2) was used to capture fluorescence images of core–shell MNs and pork rind. Mechanical properties of MNs were assessed using a universal electronic material testing machine (Instron, 5944), and heat spectra and images were recorded using thermal imaging camera (FLIR, E5xt).

### Materials

PEGDA, SA, and PVA were sourced from Sigma-Aldrich. 2Aladdin Industrial Corporation supplied TP. Pae was acquired from Shanghai Macklin Biochemical Technology Co. Ltd. Ti_3_C_2_ MXene was obtained from XFNANO Materials Tech Co. Ltd. (XFNANO). Calcein AM Cell Viability Assay Kit was purchased from Beyotime Biotechnology. Gibco provided the penicillin–streptomycin double antibiotics, pancreatic enzyme, and fetal bovine serum. We bought CCK-8 from KeyGEN BioTECH Ltd. H&E staining kit and Masson staining kit were obtained from MesGen Biotechnology. We bought antibodies from Servicebio, including IL-1, TGF-β, TNF-α, IL-6, and α-SMA. Abcam provided the PLIN1 and COL1A antibodies. All medications and reagents were utilized right away after being received and were of standard analytical levels.

### Preparation of the P-M@TP nanoparticles

Ultrathin P-M@TP with varying TP loading rates was prepared through a one-pot method. MXene was distributed in deionized water at a concentration of 0.5 mg/ml, and dopamine hydrochloride (0.2 mg/ml) was introduced. The resulting solution was divided into quintuplets, stirred for 5 min at room temperature and then mixed with Nile Red ethanol solution to form solutions with concentrations of 0, 20, 40, 80, and 120 μg/ml. The mixed solution was stirred for 30 min, pH was adjusted to 7.0 to 7.5, and the mixture was left to centrifuge at 5,000 r for 15 min. A precipitate was obtained, and the ethanol solution was used to extract free Nile Red. The Nile Red loading rate served as a metric to assess the TP adsorption ratio on P-MXene. The final P-M@TP nanomaterials were subjected to freeze-drying for 24 h and preserved at 4 °C.

### Preparation of the MNs

A solution containing Pae (0.16 mg/ml) and PVA (20%, v/v) was created, and 200 μl of this mixture was added to a custom negative electrode mold. Following a 10-min vacuum treatment, the excess solution was pipetted out and dried in a desiccator (37 °C) overnight. After drying, the cavity was filled again in the same way to ensure that the dried tip was intact. PEGDA (50%, v/v), SA (2%, w/v), 2-hydroxy-2-methylpropiophenon (HMPP; 1%, v/v), and synthetic P-M@TP were mixed and quickly vacuum-filled into PVA pretreated molds and then cured through ultraviolet irradiation for 15 s. MNs, measuring 1,000 μm in length and 450 μm in diameter, were gently peeled off the molds.

### Mechanical strength tests

MNs featuring diverse concentrations and structural compositions of PVA and other components were horizontally placed on a universal electronic testing machine with the backing layer underneath to align the tips toward the pressure transducer. The MNs were then compressed by the pressure transducer at a rate of 2 mm/min. Force measurement was initiated when the pressure transducer made initial contact with the MN, concluding when the compression force reached the testing machine’s measuring range. Data displacement curves were recorded. In the puncture experiment, fresh pig skin was selected. After cleaning the surface of the pig skin, core–shell MNs containing different colored nanoparticles were pressed onto the skin. Pressure was applied for 10 s until the needle tips pierced the skin. The skin was then placed in a thermostatic at 37 °C and irradiated with NIR for 10 min every hour to simulate MNs application. After 6 h, the MNs were removed, and the skin was observed using confocal scanning.

### NIR-triggered photothermal experiment

For the photothermal conversion experiments, MNs with different components were exposed to NIR light at 808 nm from a 5-cm distance, while the respective power levels set at 1.25, 1.5, and 1.75 W. Live temperatures of the MNs was monitored at 10-s intervals. For the open/close cycle, NIR irradiation continued up to the point where the MNs attained their peak temperature, and the following cycle commenced once the MNs had cooled to room temperature. Thermal images were captured at both the initial and final stages of the experiment on mouse skin applied with the MNs.

### Cell lines and animals

For experiments, human dermal fibroblast cell lines from the Cell Repository of the Chinese Academy of Sciences were utilized. These cells underwent incubation in complete Dulbecco’s modified Eagle’s medium [1% (v/v) of penicillin–streptomycin double antibiotics and 10% (v/v) fetal bovine serum] maintained at 37 °C and 5% CO_2_. Body weights of 20 to 25 g of female BALB/c mice were sourced from the Animal Experimentation Center of Wenzhou Research Institute and used following local ethical guidelines. The Animal Ethics Committee (Wenzhou Institute, University of Chinese Academy of Sciences) approved all animal experiments (approval WIUCAS22100901), ensuring adherence to the academy’s guidelines for animal research.

### In vitro drug release experiments

To assess the delayed release capability of the drug within the inner core layer, Cur, possessing physical and chemical properties similar to TP, was used as a substitute in the experiments. Drug-loaded MNs were immersed in buffers with varying pH. One set was immersed in PBS buffer with a pH of 7.4, while the other was placed in PBS buffer containing an equivalent amount of PVA dissolved in the outer shell layer of the MNs. The pH of the solution with the dissolved outer shell layer material was determined to be approximately 5.0. Cur release was tested at specific intervals, setting conditions at pH 5.0 and pH 7.4. The impact of NIR in the release process was separately assessed, with the NIR intervention group exposed to NIR light for 10 min/h. Each group underwent 4 parallel experiments, and drug release was recorded at 2, 4, 8, 12, 24, 48, and 72 h, respectively.

### Biosafety experiments

To sterilize the MN patches, 75% alcohol was utilized, followed by a triple rinse with PBS. MNs with varying compositions were then incubated in culture medium for 2 d. Four groups were established: The first group was a blank well plate, the second group was a blank MN patch with only the material, the third group was an MN patch spiked with P-MXene, and the fourth group was a complete MN patch with P-M@TP+Pae. The leachate was subsequently sterilized with a 200-nm syringe filter, and NIH-3T3 cell suspension was uniformly distributed among 4 groups with a concentration of 2.5 × 10^4^ cells/ml. Each group’s cell suspension (500 μl) was coincubated with the leachate from the different patch groups in 48-well plates (Corning, USA) for 72 h. Calcein acetoxymethyl ester (AM)/propidium iodide staining was performed on days 1, 2, and 3, and the cell growth status was monitored using an inverted fluorescence microscope at the same period.

### Drugs toxicity assay

A human dermal fibroblast cell suspension (2 × 10^4^ cells/ml, 200 μl) was cocultured with varying concentrations from 0.02 to 0.2 mg/ml of TP and Pae for 12 h. Six replicates were conducted in each group. The cells were incubated for 2.5 h with a new medium containing 10% CCK reagent instead of the virgin medium to facilitate color development. Subsequently, we measured the absorbance value at 450 nm. Treatment with TP resulted in significant concentration-dependent cell death.

### TP–Pae synergic therapeutic efficacy

To assess the combined impact of TP and Pae, the Chou–Talalay combination index (CI) was computed using Compusync software to calculate and identify synergistic, additive, or antagonistic interactions. TP and Pae were divided into 2 treatment groups at ratios of 4:1, 2:1, 1:1, 1:2, 1:4, and 1:5. One group received simultaneous TP and Pae treatment for 12 h, while the other had Pae treatment for 6 h, followed by simultaneous TP and Pae treatment for 6 h, simulating spatiotemporal sequential administration via MN system. Concentration gradients (0.02 to 0.2 mg/ml) of TP were established. The drug’s inhibition rate on cells was determined (4 times in parallel for each group) using CCK-8. Compusync software calculated CI values from cellular inhibition rates (CI > 1 for antagonism, CI = 1 for additivity, 0.7 < CI < 1 for slight synergism, 0.3 < CI < 0.7 for synergism, and CI < 0.3 for strong synergism), quantitatively revealing the nature and strength of drug interactions.

### Mouse model establishment and treatment

A total of 49 healthy female mice (BALB/c) were acclimatized for 1 week and subsequently randomly allocated into 7 groups. To induce skin fibrosis, each mouse received daily injections of 0.1 ml (1 mg/ml) of bleomycin (Nippon Kayaku Co. Ltd, Japan) for 4 weeks. One mouse from each group was chosen at random on the 29th day and euthanized for modeling assessment. Unmodeled healthy mice as the normal group, modeling untreated diseased mice as the model group, blank MNs with drug removal but NIR exposure (NIR group), MNs with TP added and simultaneous NIR irradiation (TP+NIR group), MNs with Pae added and simultaneous NIR irradiation (Pae+NIR group), addition of TP and Pae without NIR stimulation (TP+Pae group), and addition of TP and Pae with NIR intervention (TP+Pae+NIR group). All the groups involved in NIR stimulation received 8 min of irradiation 3 times a day. After 16 d of treatment, mice were euthanized following collection of blood from the eyes. Carefully separate the skin and organs, preserved in 4% paraformaldehyde (Beyotime Biotechnology) overnight, dehydrated using graded ethanol (70% to 100%), and vitrified with dimethylbenzene, embedded in mineral wax paraffin.

### In vivo assessment of MN toxicity

The toxicity behavior of the MNs in vivo was evaluated by monitoring the body weight of mice on day 0 (before modeling), day 28 (after modeling), and day 45 (after treatment), recording changes in body weight. Following euthanasia, we carefully separated the liver and ovaries, weighing each organ. Subsequently, these major organs were fabricated into paraffin samples, sliced into 5 μm, and subjected to H&E staining for detailed analysis.

### H&E, Masson staining, and immunofluorescence

Sections of tissue measuring 5 μm in thickness were readied for H&E and Masson staining, with subsequent statistical analysis of epidermal thickness conducted using ImageJ software. Skin tissue sections of 7 μm were used for immunofluorescence staining (TGF-β, IL-1β, α-SMA, COL1A, IL-6, TNF-α, and PLIN1), and laser confocal microscope (LSM 980 with Airyscan2) was used to observe and statistical analysis was conducted with ImageJ software.

### Statistical analysis

GraphPad Prism software was used for Statistical analysis. All data were expressed as average ± SD. Statistical significance was determined using a Student’s *t* test for comparisons between groups.

## Data Availability

All data are available in the main text or the Supplementary Materials.
