# Supplementary material for: Traditional Chinese Medicine Integrated Multifunctional Responsive Core–Shell Microneedles for Dermatosis Treatment
Source: Research (Wash D C). 2024 Jul 4;7:0420. doi: 10.34133/research.0420 (PMC11223756; doi:10.34133/research.0420)
Supplement: Supplementary 1 — Figs. S1 to S10 [file research.0420.f1.docx]

Supporting Information

Traditional Chinese medicine integrated multifunctional responsive core-shell microneedles for dermatosis treatment

Xi Luan ^1^, Xiaoxuan Zhang ^2^, Qichen Luan ^1^, Jingjing Gan ^1^, Yu Wang ^2,^* and Yuanjin Zhao ^1,2,3,^*

^1^ Department of Rheumatology and Immunology, Nanjing Drum Tower Hospital, School of Pharmacy, Clinical College of Traditional Chinese and Western Medicine, Nanjing University of Chinese Medicine, Nanjing, 210023, China;

^2^ State Key Laboratory of Bioelectronics, School of Biological Science and Medical Engineering, Southeast University, Nanjing 210096, China;

^3^ Shenzhen Research Institute, Southeast University, Shenzhen 518071, China

Email: [1146681561@qq.com](mailto:1146681561@qq.com) (Y. Wang); [yjzhao@seu.edu.cn](mailto:yjzhao@seu.edu.cn) (Y. J. Zhao)


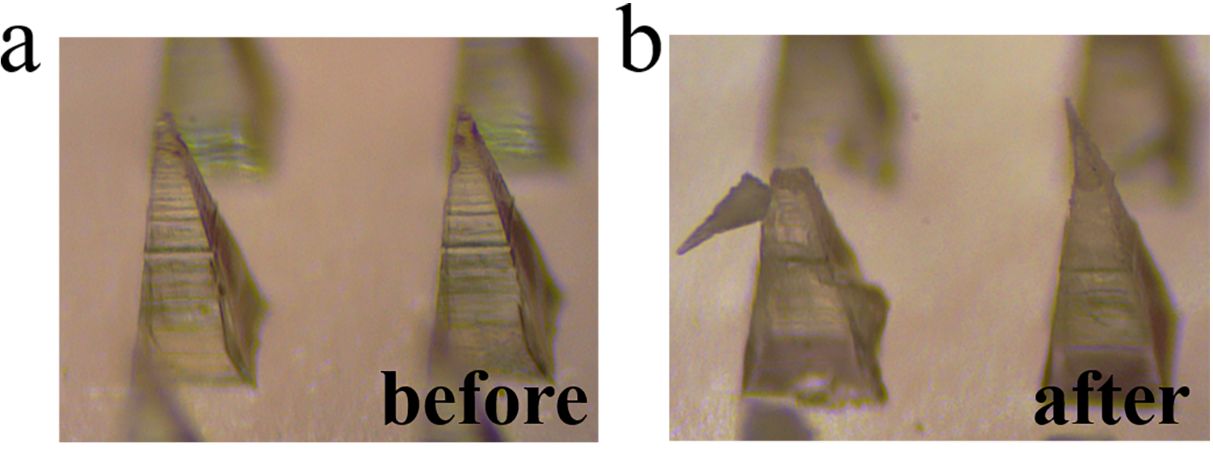


**Figure S1.** Micrograph of the tips before and after compression test.


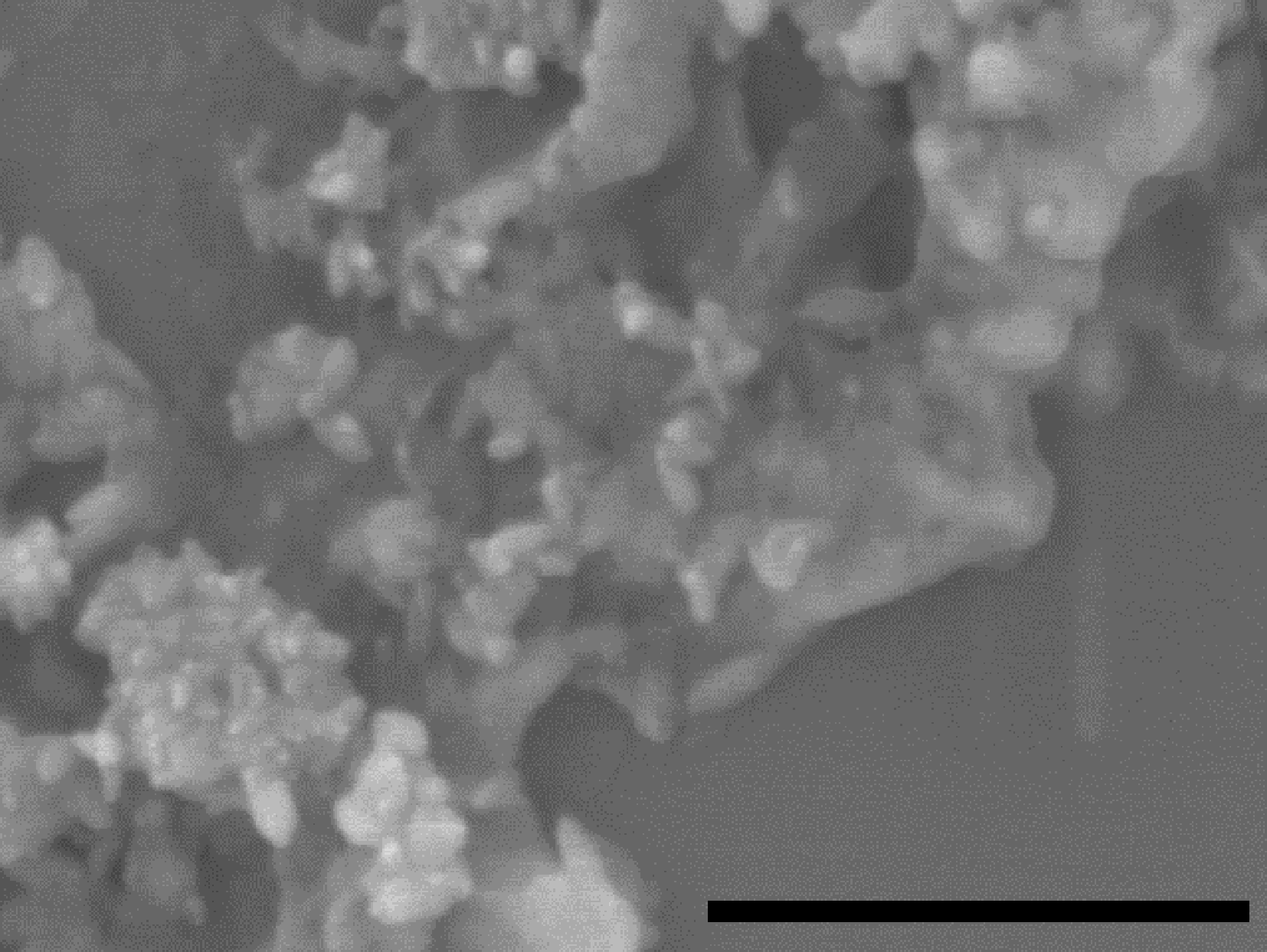


**Figure S2.** SEM photograph of PDA-Mxene@TP. The scale bar is 500 nm.


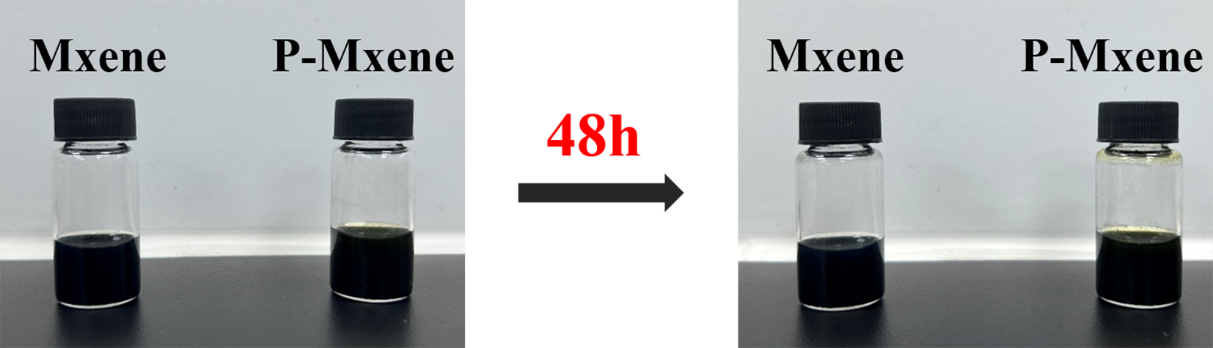


**Figure S3.** Optical image of Mxene and synthesized PDA-Mxene(P-Mxene) system before and after 48 hours of resting.


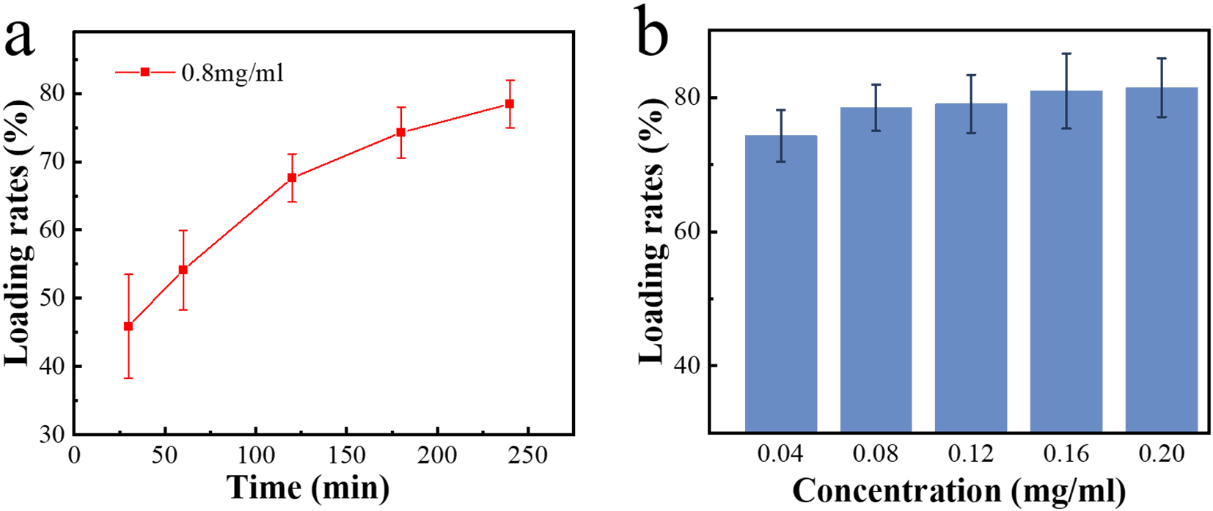


**Figure S4.** Loading rates varied of the PDA-MXene system in 4 hours(a) and in different concentrations(b).


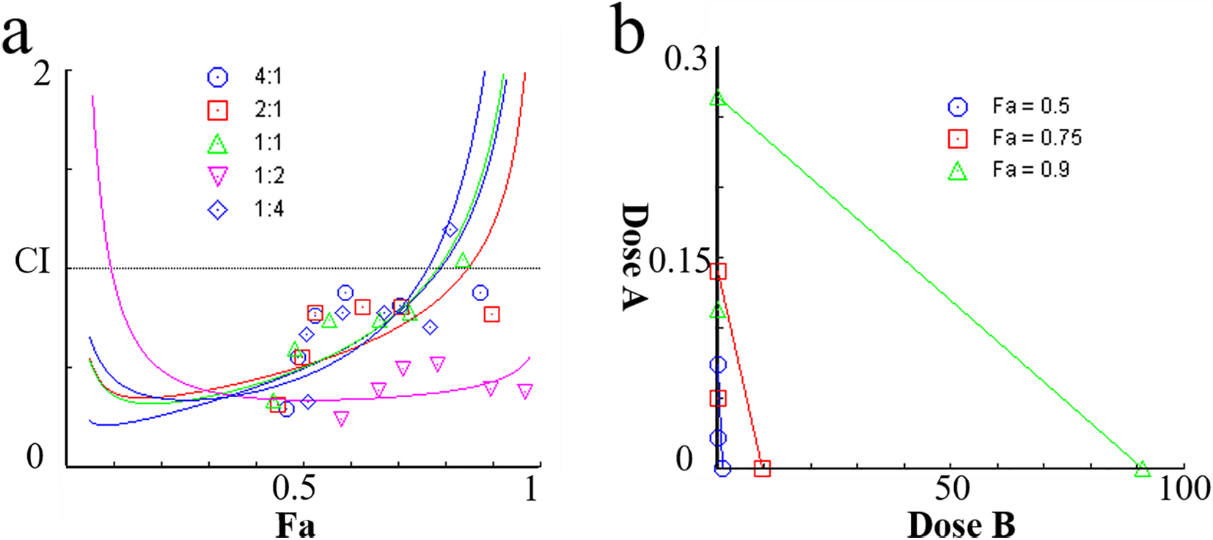


**Figure S5.** (a) Combination Index Plot when TP: Pae was on the ratio of 4:1, 2:1, 1:1, 1:2 and 1:4; (b) Isobologram for Combo 1:2 (TP: Pae [1:2]).


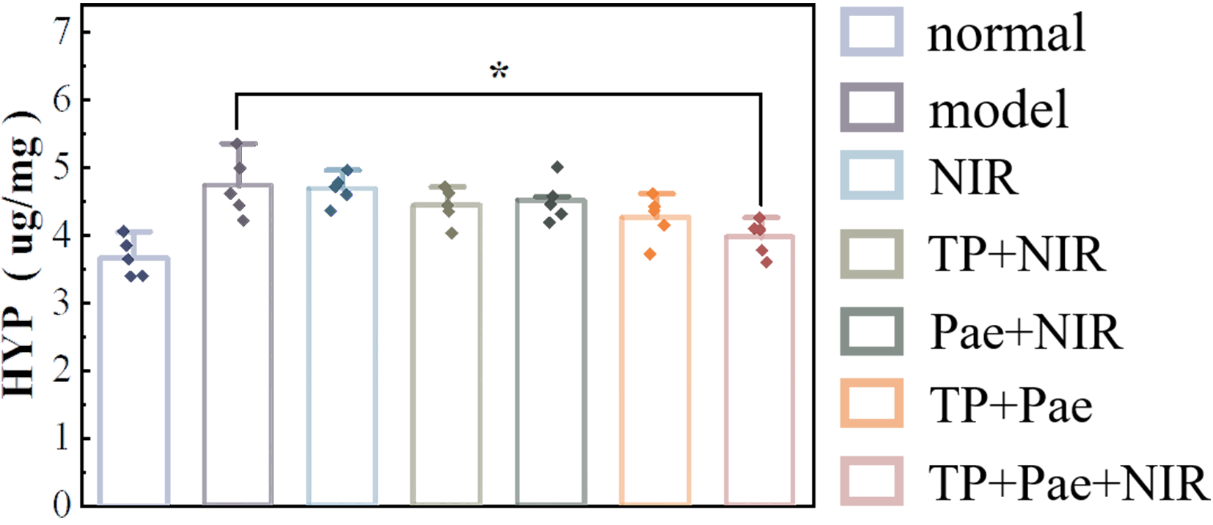


**Figure S6.** Concentration of hydroxyproline (HYP) in different groups on day 45.


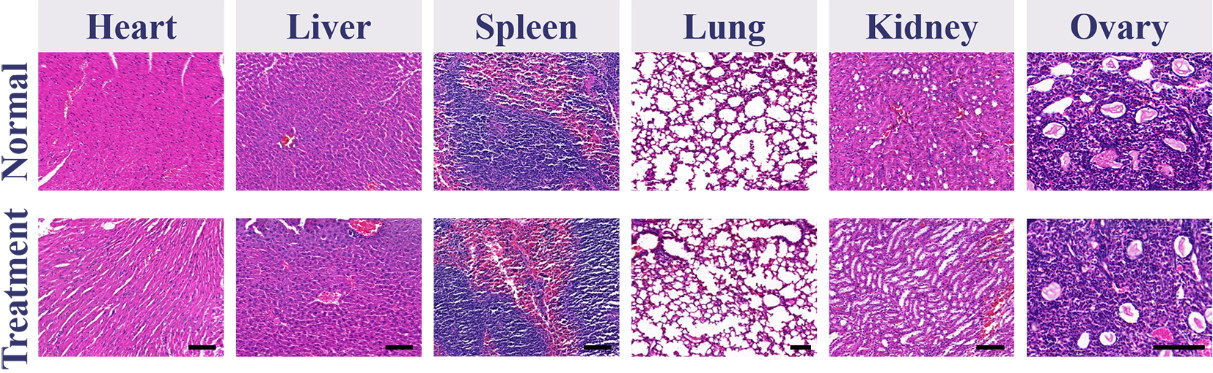


**Figure S7.** H&E staining of heart, liver, spleen, lung, kidney and ovary sections. All the scale bars are 100 μm.


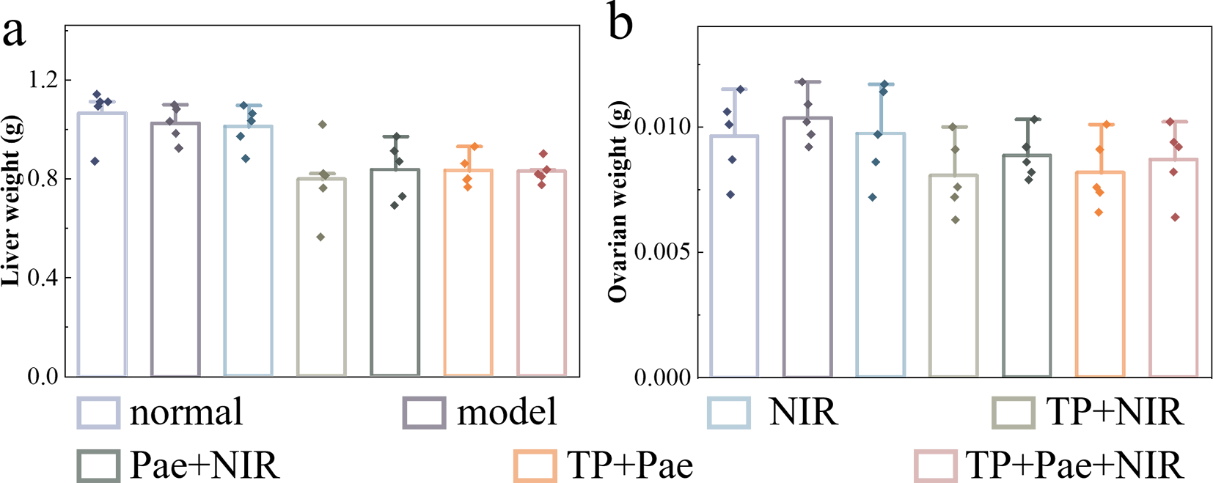


**Figure S8.** Liver and ovarian wet weights of mice in the corresponding groups after treatment. *P < 0.05.


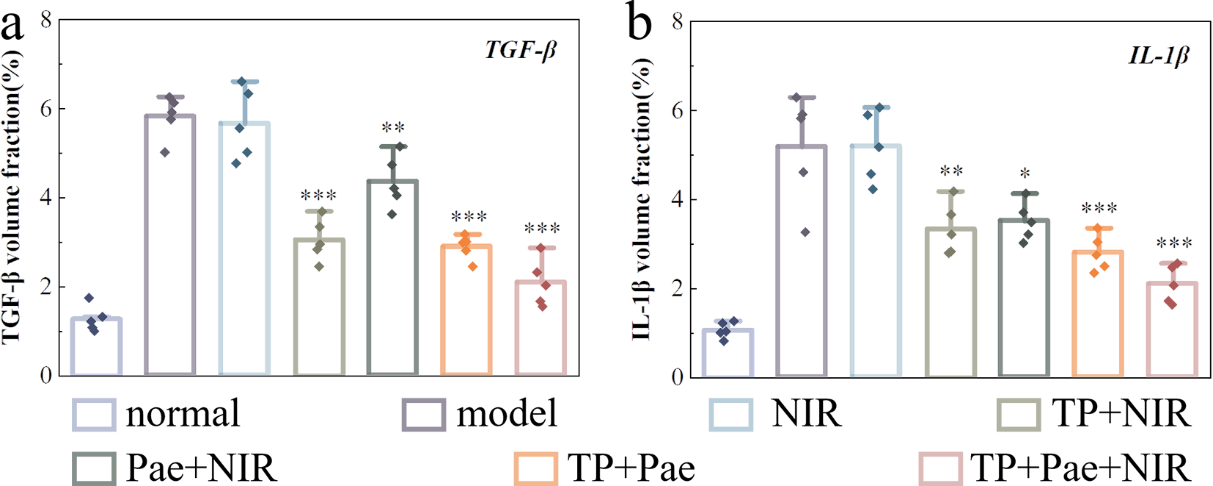


**Figure S9.** Fluorescence volume fraction statistics of TGF-β(a) and IL-1β(b). All statistical differences were compared with the model group, *P < 0.05, **P < 0.01, ***P<0.001.


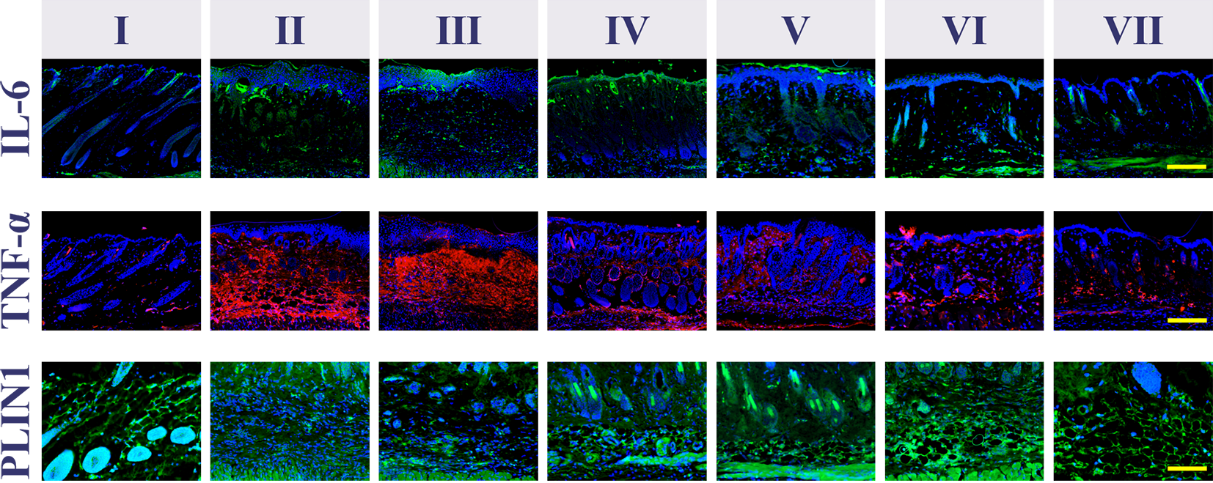


**Figure S10.** The expression of IL-6, TNF-α, and PL1N1 was detected by immunofluorescent staining in different groups (Ⅰ: normal group; Ⅱ: model group; Ⅲ: NIR group; Ⅳ: TP+NIR group; Ⅴ: Pae+NIR group; Ⅵ: TP+Pae group; Ⅶ: TP+Pae+NIR group). All the scale bars are 200 μm.
